# Mycotic aneurysms: uncommon pathogens and treatment conundrums

**DOI:** 10.1099/acmi.0.000777.v5

**Published:** 2024-08-20

**Authors:** Akshatha Ravindra, Santhanam Naguthevar, Deepak Kumar, Rengarajan Rajagopal, Pushpinder Singh Khera, Vibhor Tak, Neetha Thayil Ramankutty, Durga Shankar Meena, Naresh Midha, Gopal Krishana Bohra, Mahendra Kumar Garg

**Affiliations:** 1 Department of Medicine and Infectious Diseases, All India Institute of Medical Sciences, Jodhpur, Rajasthan, India; 2 Department of Diagnostic and Interventional Radiology, All India Institute of Medical Sciences, Jodhpur, Rajasthan, India; 3 Department of Microbiology, All India Institute of Medical Sciences, Jodhpur, Rajasthan, India

**Keywords:** blood culture, mycotic aneurysm

## Abstract

**Introduction.** Mycotic aneurysms, characterized by vessel wall dilation resulting from infections including bacteria, fungi, and viruses, are a rare but severe consequence of systemic infections. The term ‘mycotic’ was coined by William Osler to describe the first instance of a fungal-induced infected aneurysm. These aneurysms, accounting for 0.6% of aneurysms in Western countries, carry a higher risk of rupture compared to uninfected aneurysms. While the femoral artery, aorta, and intracranial arteries are commonly affected, pathogens causing mycotic aneurysms vary across regions. Diagnostic challenges arise from nonspecific symptoms such as fever, and discomfort. To prevent the substantial morbidity and mortality associated with mycotic aneurysms, timely identification and treatment are paramount. We present a case series highlighting mycotic aneurysms caused by some rare pathogens – *Salmonella Paratyphi A, Streptococcus pneumoniae*, and *Pseudomonas aeruginosa*.

**Methods.** This case series involves three patients diagnosed with mycotic aneurysms due to unusual pathogens. We describe each patient’s clinical presentation, medical history, physical examination findings, laboratory results, imaging studies, and the diagnostic process leading to the identification of the causative pathogens.

**Results.** The first patient is a 70-year-old gentleman who presented with a ruptured infra-renal abdominal aortic pseudoaneurysm caused by *Salmonella Paratyphi A*. The second patient is a 66-year-old gentleman with a *Streptococcus pneumoniae*-associated descending thoracic aortic pseudoaneurysm. The third patient is a 70-year-old gentleman with a ruptured descending thoracic aortic aneurysm with an occult aorto-oesophageal fistula due to *Pseudomonas aeruginosa* infection. The description highlights unique clinical features, laboratory findings, imaging results, and the management approaches undertaken in each patient.

**Conclusion.** Mycotic aneurysms, pose diagnostic challenges due to their nonspecific symptoms. Early identification and intervention are essential to mitigate the severe complications associated with these aneurysms. The presented cases underscore the need for a comprehensive approach to diagnosis and management, ensuring optimal outcomes for patients affected by mycotic aneurysms.

## Data Summary

No new data, tools, software or code has been generated or is required for our work to be reproduced.

## Introduction

A mycotic aneurysm is defined as the dilatation of a vessel wall due to infection, which can be a bacteria, fungus, or virus. The term ‘mycotic’ is a misnomer, as it was initially named by William Osler to describe the first case of infected aneurysm caused by fungus [1,3]. It is a rare but severe consequence of systemic infection that frequently poses several diagnostic and therapeutic problems. This accounts for about 0.6% of all aneurysms in Western countries and is associated with a greater risk of rupture than uninfected aneurysms such as those due to arteriosclerosis [1,2, 4]. The vessels most frequently implicated are the femoral artery, followed by the aorta, and subsequently, the intracranial and visceral arteries (e.g., superior mesenteric, splenic) [1,2]. The progression of this condition is marked by gradual expansion, which ultimately gives rise to the formation of a pseudoaneurysm, followed by rupture, haemorrhage, the onset of sepsis, and subsequent development of multiorgan failure [1,5]. The etiological agents in the Western countries include *Staphylococcus aureus* (28%), *Salmonella* spp*.* (15%), and *Pseudomonas aeruginosa* (10%), but *Salmonella* is the most common pathogen in most Asian countries [1,2, 5]. Fever, pulsatile mass, local discomfort, and arterial site inflammation (back pain in the aorta, headaches in cerebral arteries) are the most typical signs [1,2, 5]. Because these symptoms are non-specific, many individuals are classified as having a fever of unknown origin and go undetected until they develop severe symptoms of sepsis, thrombosis, haemorrhage, or rupture [6]. To reduce the severe morbidity and mortality associated with this illness, timely identification and treatment are required. We present a case series of mycotic aneurysms caused by rare pathogens – *Salmonella Paratyphi A, Streptococcus pneumonia*, and *Pseudomonas aeruginosa* highlighting the subacute presentation, diagnostic challenges, and management complexities associated with mycotic aneurysms.

## Methods

A prospective cross-sectional analysis of hospital records was performed between the period from July 2021 to July 2023 after approval from institutional ethics committee. All patients with definite/possible mycotic aneurysm diagnosed based on clinico-radiological criteria were included in the final report. All diagnosed cases of mycotic aneurysm patients had three sets of blood cultures drawn from three separate venepuncture sites, 1 h apart before initiation of antibiotics. Data on demographics, risk factors, clinical signs and symptoms, echocardiography findings, microbiological aetiology, complications, treatment, and outcomes were collected.

## Results

A total of three patients were diagnosed with mycotic aneurysms during the study period. Of these, *Salmonella* Paratyphi A, *Streptococcus pneumoniae*, *Pseudomonas aeruginosa* were isolated in one patient each. The clinical course of the three patients are described below.

### Patient 1

A 70-year-old gentleman presented to the emergency department with subacute onset of periumbilical pain and high-grade fever with chills and rigours for 20 days. The pain was dull aching, dragging type with no radiation. He denied any history of cough, dyspnoea, chest pain, or weight loss. He had no significant co-morbidities or medical history. At the time of hospitalization, he was febrile – 103.5 °F. His vitals were stable with blood pressure of 110/60 mmHg, pulse was 80 min^−1^, respiratory rate of 18 min^−1^, oxygen saturation of 97% on room air. He had some discomfort on palpation of the abdomen with mild hepatosplenomegaly. Other systems examination was unremarkable.

Initial investigation showed a haemoglobin of 11.6 g dl^−1^, hematocrit of 43.3%, total leucocyte count of 16 660 cells/mcl (differential count of neutrophils (N) – 84%/lymphocytes (L) – 9%/monocytes (M) – 5.9%/eosinophils (E) – 0.1%). His inflammatory markers were raised (ESR-79 mm/h, CRP −138 mg l^−1^, procalcitonin-2.69 ng ml^−1^). His renal function tests and liver function tests were deranged (urea of 39 and creatinine-1.91 mg dl^−1^, eGFR-64 ml min^−1^ and albumin of 2.9 mg dl^−1^). Ultrasonography of his abdomen confirmed hepatosplenomegaly with altered renal cortico-medullary differentiation. Paired blood cultures were sent, which showed growth of *Salmonella Paratyphi A* by MALDI TOF MS in both the samples. The antimicrobial susceptibility test by microbroth dilution showed susceptibility to beta-lactams including ampicillin, ceftriaxone, and cotrimoxazole. He was administered ceftriaxone 1 g intravenously every 12 hours based on the bacterial culture sensitivity after test dose. He had persistent fever, and repeat blood cultures were sent, which revealed persistent bacteraemia. The ceftriaxone dose was increased to 2 g IV every 12 h. To look for the cause of persistent bacteraemia, transthoracic echocardiography was performed, which showed no evidence of infective endocarditis.

**Fig. 1.**
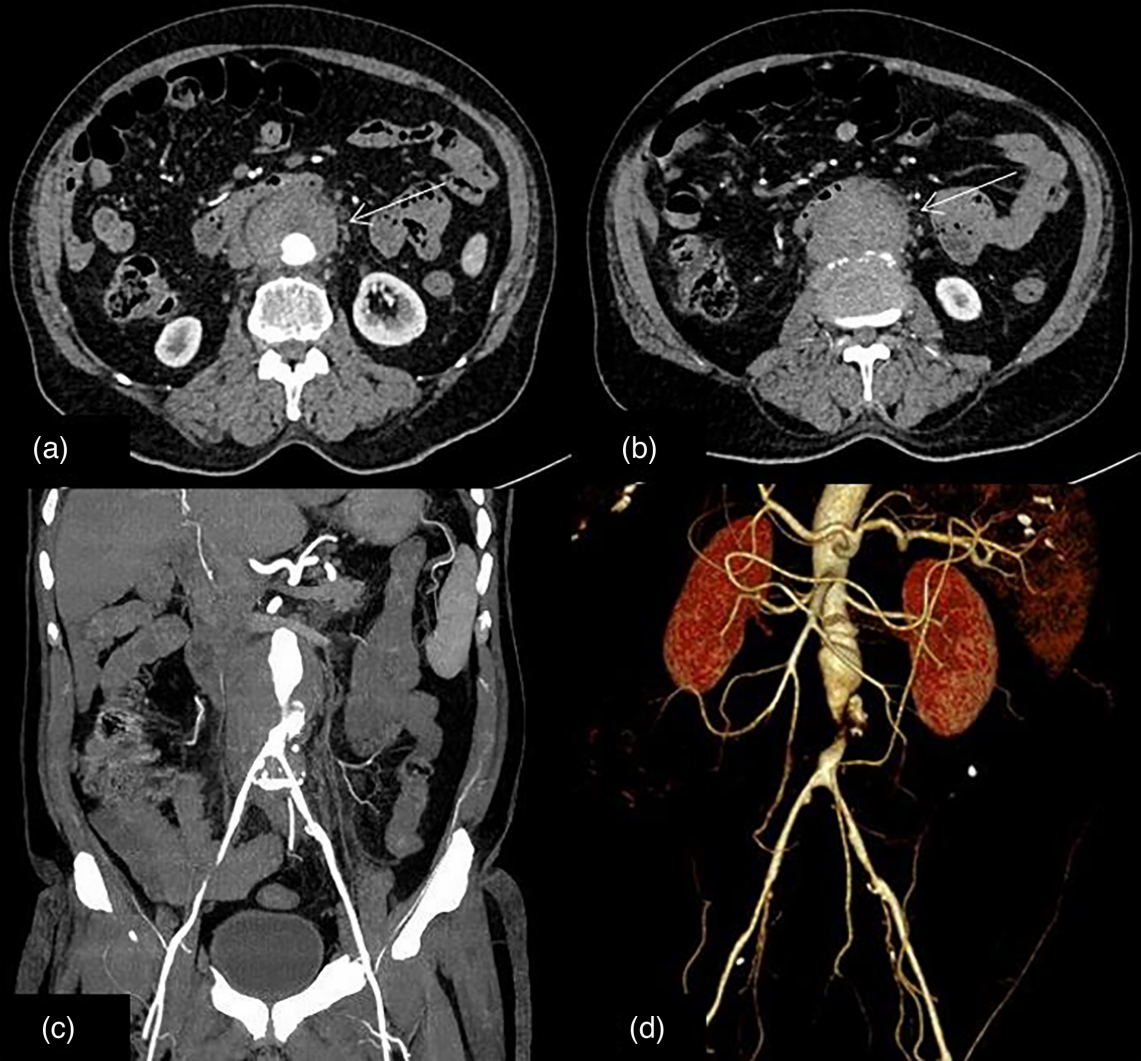
CT abdominal angiograms in a 70-year-old gentleman showing a ruptured infra-renal abdominal aortic pseudoaneurysm (white arrow in a, and c). His blood cultures were positive for *Salmonella paratyphi A*. Repeat CT angiogram after antibiotic therapy showed thrombosis of the pseudoaneurysm (white arrows in b, and d).

CT abdominal angiography was performed, which revealed ruptured infrarenal abdominal aortic pseudoaneurysm with surrounding hematoma (Fig. 1). The final diagnosis of ruptured infra renal abdominal aortic pseudoaneurysm and acute kidney injury due to septicemia caused by *Salmonella Paratyphi A* was made. The patient’s general condition had improved with reduced fever spikes and recovery renal function test. As with any case of mycotic aneurysm with rupture, we planned for elective endovascular repair of his aortic aneurysm, but the patient did not provide consent for any intervention. The patient was managed with antimicrobial therapy with IV ceftriaxone 2 g every 12 h for 6 weeks. A repeat CT angiogram showed complete thrombosis of the previous aortic lesion and abdominal aorta (Fig. 1). After 6 weeks, the patient was asymptomatic, repeat cultures were sterile. The patient was lost to follow-up after discharge.

### Patient 2

A 66-year-old gentleman presented to the emergency department with abdominal pain radiating to the back for 20 days, which had increased in the last 5 days. He also had a history of constipation and loss of appetite. He denied any history of cough, dyspnoea, chest pain, or weight loss. He had no significant co-morbidities or medical history. At the time of hospitalization, he was febrile with temperature of 101.2 °F. His vital signs were stable. On physical examination, His abdomen was soft, with tenderness in the left lower quadrant. No pulsatile mass was present.

Initial investigation showed a haemoglobin of 10.6 g dl^−1^, hematocrit of 43.3%, total leucocyte count of 17680 cells mcl^−1^ (differential count of N-79%/L-11%/M-9%/E-0.1%). His inflammatory markers were raised (ESR-120 mm h^−1^, CRP −188 mg l^−1^, procalcitonin-1.06 ng ml^−1^).

**Fig. 2.**
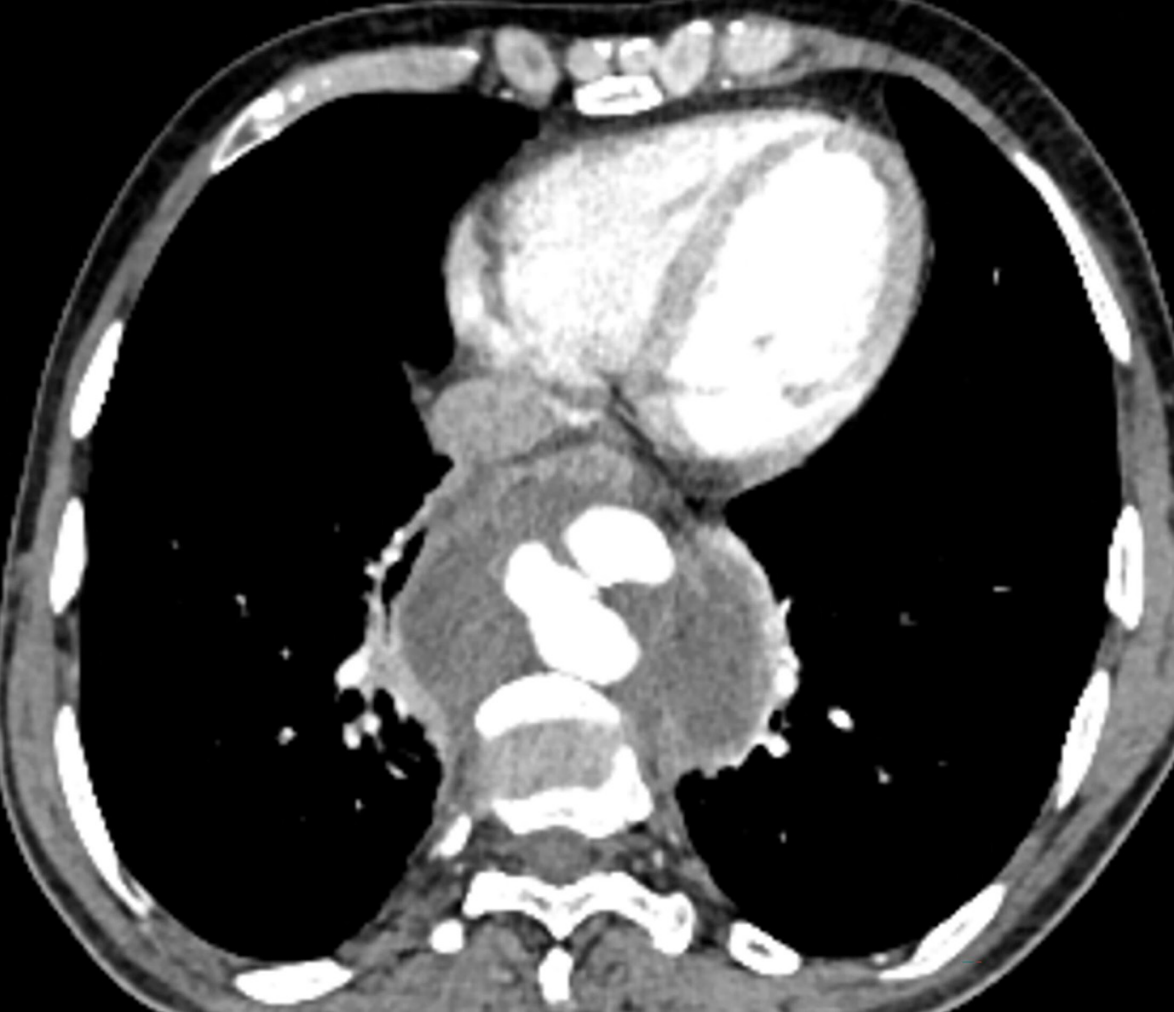
CECT thorax in a 66-year-old gentleman showing a large thoracic aortic pseudoaneurysm, with surrounding hematoma. His blood cultures were positive for *Streptococcus pneumoniae*.

With a clinical working diagnosis of possible tuberculosis due to abdominal symptoms and the high prevalence of tuberculosis in our region, CECT thorax and abdomen was done, which revealed a large thoracic aortic pseudoaneurysm, with surrounding hematoma and mediastinal lymphadenopathy (Fig. 2). Paired blood cultures were sent, which showed growth of *Streptococcus pneumoniae by* MALDI TOF MS, which was susceptible to penicillin with MIC of 0.06 mcg ml^−1^ by microbroth dilution in both the cultures and ampicillin 2 g every 6 h was started intravenously based on the bacterial culture sensitivity after test dose. Aortogram revealed pseudoaneurysm arising from descending thoracic aorta, measuring 4.2×4.6×2.5 cm, across D9 to D11 level. A final diagnosis of ruptured descending thoracic aortic pseudoaneurysm due to septicemia secondary to *Streptococcus pneumonia*e was made. He was then planned for thoracic endovascular aortic aneurysm repair (TEVAR) under GA. Right femoral arteriotomy was done followed by which a stent graft (Endurant 28×28J 82 mm) was placed across the neck of the pseudoaneurysm in descending thoracic aorta. However, due to inadequate proximal landing zone, a second stent graft (Valint Captiva Thoracic aortic stent graft 30×30×117 mm) was deployed. Post-stenting angiogram revealed no opacification of the pseudoaneurysm and normal opacification of mesenteric vessels and adequate proximal and distal aneurysm neck coverage. He withstood the procedure well, with no post-procedural complications. A repeat CT angiogram after 2 weeks revealed no contrast opacification of the aneurysm with normal contrast flow through the stent graft in DTA with surrounding mediastinal hematoma. Repeat cultures were sent and were sterile. He was discharged on Tab.amoxicillin 1 g every 8 h for 4 weeks. After 6 weeks, he was asymptomatic, repeat cultures were sterile.

### Patient 3

**Fig. 3.**
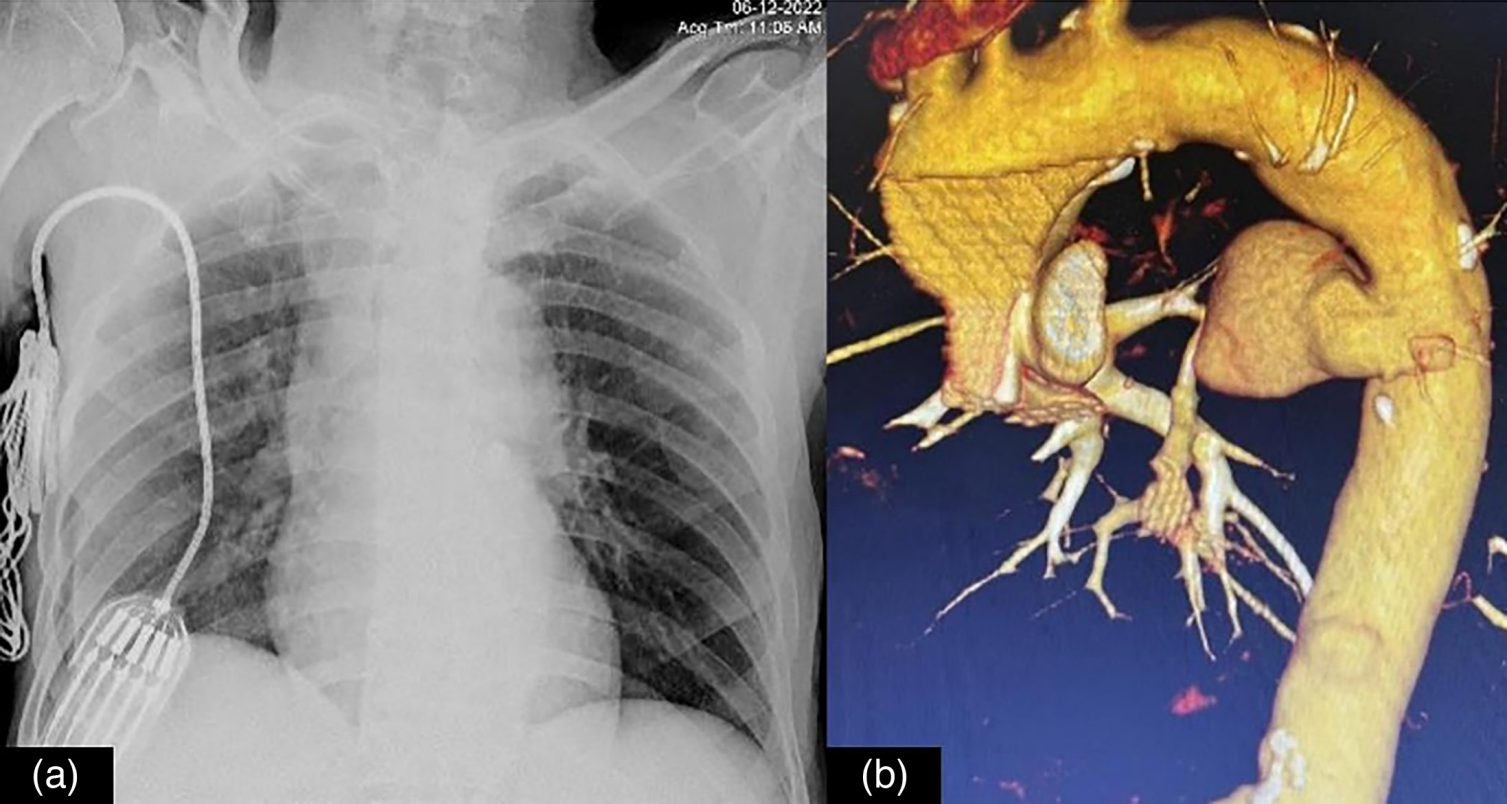
Chest radiograph in a a 70-year-old gentleman showing mediastinal widening (**a**). His aortogram subsequently revealed a rupture descending thoracic aortic pseudoaneurysm (**b**). His blood cultures were positive for *Pseudomonas aeruginosa*.

A 70-year-old gentleman presented with multiple episodes of hematemesis and melena for 15 days. He had undergone emergency coronary artery bypass grafting with saphenous vein graft to the left descending artery elsewhere 3 months prior. Four weeks after the CABG, he had purulent discharge from the leg wound, for which he was given antibiotics for a few days in a local hospital, after which the wound healed. He then developed low grade fever, which was relieved on medications including paracetamol and multiple oral antibiotics (exact documents not available) over the next 2 months. Subsequently, He started passing dark stools, and in the next few days, had two–three episodes of bloody vomiting (around 30–40 ml per episode). He also experienced sharp shooting pain in chest for which he was brought to the hospital. On examination, he was tachycardic (120 min^−1^), and hypotensive (BP – 80/60 mmHg). His chest x-ray showed widening of the mediastinum. CT angiography was performed which revealed a pseudoaneurysm of descending thoracic aorta measuring ~3.4×2.8 cm at the level of thoracic vertebrae – D6–D7 vertebra with surrounding mediastinal hematoma and the ill-defined posterior wall of the oesophagus with hyper-dense content in the oesophagus and stomach likely due to an occult aorto-oesophageal fistula. The pseudoaneurysm was directed anteromedially and causing mass effect over the oesophagus and left atrium in the form of anterior displacement (Fig. 3).

Blood cultures were drawn and he was initiated on piperacillin–tazobactam intravenously. He was planned for emergency thoracic aortic endovascular repair. Post stent-graft deployment, he developed multiple successive bouts of massive hematemesis. A repeat angiogram showed diffuse oozing through the graft due to very low platelets and a deranged clotting profile. He succumbed due to massive hemetemesis. His blood culture reports later revealed growth of *Pseudomonas aeruginosa*.

## Discussion

### Epidemiology and clinical spectrum

Mycotic aneurysms are rare though the exact incidence is unknown. Few studies have reported an incidence around 0.7–3% of all aneurysms in Western countries, and their potential to lead to rupture and severe outcomes is higher when compared to non-infected aneurysms [1,2]. The aneurysms predominantly manifest in the femoral artery, followed by the aorta, intracranial, and visceral arteries. Interestingly, etiological agents vary geographically, reflecting differing pathogen prevalence. The predominant pathogens encompass *Staphylococcus aureus* along with *Salmonella* (15%). Rare cases of other micro-organisms that have been implicated include *Treponema pallidum, Mycobacterium* spp., *Pseudomonas aeruginosa, Listeria, Klebsiella* etc as in our cases [1,5].

### Diagnostic quandaries and clinical presentation

The clinical presentation of mycotic aneurysms is far from straightforward, characterized by fever, discomfort, and arterial inflammation [1,2, 4]. However, these non-specific symptoms often lead to misdiagnosis as fever is of an unknown origin [6]. This diagnostic challenge is exacerbated by the tendency of these lesions to remain undetected until they develop severe complications, such as sepsis, thrombosis, haemorrhage, or rupture [1]. Timely identification and treatment are crucial to curbing the substantial morbidity and mortality associated with this condition.

### Challenges in management

The management of mycotic aneurysms is multifaceted, involving antimicrobial therapy and often necessitating surgical intervention [1,2]. Notably, surgical repair is complicated by the risk of rupture [7]. Elective endovascular repair, as depicted in case 1, is a viable option to mitigate this risk. However, patient reluctance to undergo surgical intervention can influence treatment decisions. Moreover, achieving sterile cultures and resolution of symptoms post-treatment, as seen in case 2, signifies successful management, underlining the significance of both antimicrobial therapy and procedural interventions.

### Mycotic aneurysm due to non-typhoidal salmonella

Non-typhoidal salmonella (NTS) usually causes mild self-limiting gastroenteritis. Transient bacteraemia occurs in 2–8% of patients [8,9]. This can later involve the heart and arterial walls leading to endocarditis, pericarditis, aortitis, etc. Mycotic or infected aneurysm due to NTS is quite rare occurring in 9–40%, the most common site involved is the infrarenal abdominal aorta followed by the thoracic and suprarenal abdominal aorta [1,9, 10]. The mechanism proposed for mycotic aneurysm is bacterial seeding on the damaged atherosclerotic intima and subsequent aneurysm. Given the absence of significant comorbidities in our patient, the source could be a recent ingestion of contaminated food or water, leading to systemic infection and seeding over the existing athersosclerotic intima and localization in a vascular lesion. One must suspect endovascular infection in NTS when there is persistent fever, relapsing bacteraemia or remitting sepsis is present [11]. NTS vascular infection (NTSVI) score was proposed to identify the patients with risk for endovascular infection which include male sex, hypertension, coronary artery diseases, serogroup C1 infection (+1), while malignancy and immunosuppressive therapy are associated with lower risk (−1) [12]. Other complications of NTS bacteraemia can be vertebral osteomyelitis, gastrointestinal bleeding due to fistula formation. Catastrophic consequences including mortality can occur in cases where these complications are not addressed [13]. Diagnosis can be made based on imaging including a CT scan of the abdomen, which may demonstrate an infected aneurysm as periaortic soft tissue density with rim enhancement. Our patient had risk factors for NTS endovascular infection with a NTSVI score of 2, had persistent bacteraemia, and relapsing fever. Active efforts were done to look for complications, which helps in earlier diagnosis and management with appropriate antibiotics and surgery. The treatment of choice is third-generation cephalosporins, but the corner stone of management of mycotic aneurysms remains surgery [9,11, 14]. In a study done by Soravia-Dunand *et al*. who reviewed 140 cases of aortitis, 96% mortality occurred when only medical treatment was provided, while combination of surgery and medical treatment was associated with lower mortality (40%) [14]. Surgical treatment consists of debridement of infected tissues with endovascular stent graft. Our patient refused any surgical management and hence was managed with only antibiotics and was doing well after 6 weeks of therapy. The duration of therapy is not defined, and as a general rule for endovascular infection, a minimum of 6 weeks is recommended. Lifelong therapy may be required in certain patients unfit for surgery, and with relapsing bacteraemia [8,9, 14].

### Mycotic aneurysm due to *Streptococcus pneumoniae*

While *Streptococcus pneumoniae* is a well-known pathogen responsible for respiratory and invasive infections, its involvement in mycotic aneurysms is not widely reported [7,15]. Few cases have been reported in the literature in which the patients can develop aortitis, without having features of pneumonia, as in our case [7]. The patient’s clinical presentation with abdominal pain, back pain, and fever was indicative of an underlying pathology, yet the non-specific nature of these symptoms might have led to initial confusion or misdiagnosis. It was only through comprehensive imaging, in this case, CECT thorax and abdomen, that the presence of a large thoracic aortic pseudoaneurysm was revealed. The early identification of the causative agent, in this case, *Streptococcus pneumoniae*, was crucial for tailoring appropriate antimicrobial therapy. We hypothesize that aspiration of oropharyngeal secretions, common in older adults, leading to bacteremia and seeding of the aortic wall. When blood cultures prove ineffective in isolating the organism, 16 s RNA PCR becomes notably valuable [7]. Rising resistance to beta-lactam and other antibiotics emphasizes the importance of factoring in geographic location and epidemiological context when selecting empirical treatment strategies for culture-negative patients. Most of the cases of *Streptococcus pneumonia* aneurysms were treated with surgical repair and in a few patients who were managed medically were given lifelong prophylaxis or was associated with fatal outcome [7,15,17]. The duration of therapy varied in different cases, with most receiving therapy for up to 6 weeks [7,15,17].

### Mycotic aneurysm due to *Pseudomonas aeruginosa*

*Pseudomonas aeruginosa* is one of the rare causes of mycotic aneurysm. It likely that our patient acquired this during postoperative care, either from the infected leg wound or during invasive procedures, leading to bacteremia and vascular infection. Most of the cases associated with *Pseudomonas* was in association with solid organ transplant or secondary to the surgical procedure such as grafts [18,22]. Case 3 highlights the importance of considering vascular complications in post-surgical patients, particularly when they present with symptoms seemingly unrelated to the cardiovascular system. In this patient, the widening of the mediastinum seen on the chest x-ray and subsequent CT coronary angiography were pivotal in revealing the presence of the pseudoaneurysm. *Pseudomonas aeruginosa* is known for its virulence and propensity to cause severe infections, particularly in immunocompromised patients or those with significant medical history [23]. In this case, the patient’s history of coronary bypass surgery and subsequent wound infection might have facilitated the entry of this opportunistic pathogen into the bloodstream and the aortic wall.

## Conclusion

The presented case series unveils the intricacies of mycotic aneurysms, highlighting the diagnostic challenges and therapeutic complexities posed by these rare vascular anomalies. By showcasing the diverse clinical presentations and management approaches, this case series contributes to the collective understanding of mycotic aneurysms. Ultimately, the lessons gleaned from these cases reinforce the importance of prompt identification, tailored treatment, and interdisciplinary collaboration in addressing this uncommon yet high-stakes' condition.

## Abbreviations

CABG: coronary artery bypass grafting
CECT: contrast-enhanced computed tomography
CRP: C-reactive protein
CT: computed tomography
DTA: descending thoracic aorta
eGFR: estimated glomerular filtration rate
ESR: erythrocyte sedimentation rate
GA: general anesthesia
IV: intravenous
MALDI-TOF MS: matrix-assisted laser desorption Ionization time-of-flight mass spectrometry
mcl: microliter
MIC: minimum inhibitory concentration
Tab: tablet
TEVAR: thoracic endovascular aortic repair
